# The Antitumor Peptide CIGB-552 Increases COMMD1 and Inhibits Growth of Human Lung Cancer Cells

**DOI:** 10.1155/2013/251398

**Published:** 2013-01-16

**Authors:** Julio R. Fernández Massó, Brizaida Oliva Argüelles, Yelaine Tejeda, Soledad Astrada, Hilda Garay, Osvaldo Reyes, Livan Delgado-Roche, Mariela Bollati-Fogolín, Maribel G. Vallespí

**Affiliations:** ^1^Department of Genomic, Center for Genetic Engineering and Biotechnology, Cubanacan, P.O. Box 6162, 10600 Havana, Cuba; ^2^Pharmaceutical Department, Laboratory of Cancer Biology, Center for Genetic Engineering and Biotechnology, Cubanacan, P.O. Box 6162, 10600 Havana, Cuba; ^3^Cell Biology Unit, Institute Pasteur of Montevideo, Mataojo 2020, 11400 Montevideo, Uruguay; ^4^Synthetic Peptide Group, Physical Chemistry Department, Center for Genetic Engineering and Biotechnology, Cubanacan, P.O. Box 6162, 10600 Havana, Cuba; ^5^Center of Studies for Research and Biological Evaluations, Pharmacy and Food Sciences College, University of Havana, 19250 Havana, Cuba

## Abstract

We have demonstrated that the peptide L-2 designed from an alanine scanning of the *Limulus*-derived LALF32-51 region is a potential candidate for the anticancer therapy and its cell-penetrating capacity is an associated useful property. By the modification in the primary structure of L-2, a second-generation peptide (CIGB-552) was developed. However, the molecular mechanism underlying its cytotoxic activity remains partially unknown. In this study, it was shown that CIGB-552 increases the levels of COMMD1, a protein involved in copper homeostasis, sodium transport, and the NF-**κ**B signaling pathway. We found that CIGB-552 induces ubiquitination of RelA and inhibits the antiapoptotic activity regulated by NF-**κ**B, whereas the knockdown of COMMD1 blocks this effect. We also found that CIGB-552 decreases the antioxidant capacity and induces the peroxidation of proteins and lipids in the tumor cells. Altogether, this study provides new insights into the mechanism of action of the peptide CIGB-552, which could be relevant in the design of future anticancer therapies.

## 1. Introduction

In previous work, a peptide-based approach was used to identify peptides devoid of LPS-binding capacity from LALF residues 32–51. Two peptides (L-2 and L-20) lost their ability to bind LPS and exhibited a differential cytotoxic activity, although a similar cell penetrating capacity was demonstrated for both peptides [1]. We introduced a chemical modification in the primary structure of the peptide L-2 to improve the biological activity and specificity. The chemical modification included the substitution of a natural amino acid residue by an unnatural amino acid (D-configuration) and blocked N-terminal by acylation. This modification led to the development of a second-generation peptide (CIGB-552) with increased cytotoxic activity on murine and human tumor cells [2]. Although the antitumor effects of the peptides involve an increase in the apoptosis and a negative regulation of cell-cycle progression, little is known regarding this mechanism of action.

In this study, two complementary approaches were used: a yeast two-hybrid search for molecules that specifically interact with the peptides and a pull-down technique to validate the interaction. COMMD1 was identified as a peptide-binding protein. Furthermore, the specificity peptide/COMMD1 complexes were corroborated by related synthetic peptides with a differential cytotoxic activity (L-2, L-20, and CIGB-552) in cells expressing endogenous COMMD1. 

COMM domain—containing 1 (COMMD1), the first COMMD family member to be identified, is a pleiotropic factor that participates in multiple processes, including copper metabolism, sodium excretion, inflammatory responses, and adaptation to hypoxia [3–6]. A growing body of data suggests that COMMD1 is associated with a multimeric E3 ubiquitin ligase complex and regulates the stability of proteins such as NF-*κ*B subunits, ATP7B, and HIF-1-*α* [7–11]. In addition to its physiological roles, HIF participates in the pathophysiology of several disorders, including cancer, in which enhanced HIF activity is associated with tumor growth, neovascularization, local invasion, metastatic disease, and poor clinical outcomes [12]. While under physiological conditions NF-*κ*B plays critical roles in inflammatory responses and cellular survival to stress, the activation of NF-*κ*B has also a frequent occurrence in cancer. In particular, the ability of NF-*κ*B to promote the expression of various antiapoptotic factors is thought to play a major role in the survival of cancer cells [11]. 

Consistent with the notion that COMMD1 functions in multiple cellular pathways involved in the survival of cancer cells, it has been demonstrated that the decreased COMMD1 expression in human cancer correlates with a more invasive tumor phenotype [13]. It is reported in this study that CIGB-552 increases the levels of the protein COMMD1 and negatively regulates the anti-apoptotic activity of NF-*κ*B. Furthermore, CIGB-552 induces an imbalance in the antioxidant/prooxidant balance in cancer cells that promotes the peroxidation of proteins and lipids. These findings have relevance for the design of anticancer agents that act by targeting COMMD1 to inhibit NF-*κ*B activity.

## 2. Materials and Methods

### 2.1. Peptides Synthesis

Peptides were synthesized on a solid phase and purified by reverse-phase-high-performance liquid chromatography to >95% purity on an acetonitrile/H_2_O-trifluoracetic acid gradient and confirmed by ion-spray mass spectrometry (Micromass, Manchester, UK). Lyophilized peptides were reconstituted in phosphate-buffered saline (PBS) for experiments* in vitro*. The sequences of peptides used were L-2: HARIKPTFRRLKWKYKGKFW; L-20: HYRIKPTFRRLKWKYKGKFA and CIGB-552 second-generation peptide Ac-HARIK**p**TFRR**l**KWKYKGKFW where proline and leucine were substituted by D-amino acid; and N-terminal blocked by acylation. 

### 2.2. Yeast Two-Hybrid Screening

Oligonucleotides with the sequences corresponding to peptides L-2 and L-20 were synthesized and cloned in-frame into pGBKT7 yeast two-hybrid vector (Table 1). The recombinant clones pGBKT7-L2 and pGBKT7-L20 were verified by DNA sequence and subsequently transformed into yeast strain AH109. 

A matchmaker pretransformed liver cDNA library in Y187 (Clontech) was used to identify the protein interactions of L-2. Briefly, 5 × 10^8^ AH109 cells containing the plasmid pGBKT7-L2 were grown and matted with 5 × 10^8^ Y187 cells containing the cDNA library from human liver and transferred to minimal medium plates SD-Trp-Leu-His-Ade and grown at 30°C 7 days. The positive colonies were grown in Trp-Leu medium and the plasmids recovered and transformed into DH10B cells. Each individual clone was transformed in Y187 strain and the interaction verified by matting with the strain AH109 transformed with the plasmids pGBKT7, pGBKT7-L2, and pGBKT7-L20. The positive clones were sequenced and their sequences were analyzed using BLAST [14]. 

COMMD1 subclones N-terminus containing 6–110 amino acids and C-terminus containing 111–190 amino acids (nucleotides 18–332 and 333–570, resp.) were cloned in-frame into a two-hybrid yeast vector containing the GAL4 activation domain (pGADT7). Each COMMD1 subclone was transformed into Y187 strain and the interaction verified by matting with the AH109 strain transformed with the plasmids pGBKT7, pGBKT7-L-2, and pGBKT7-L-20.

### 2.3. Chemicals Reagents

The following chemicals and reagents used were from the indicated companies: RPMI 1640 (Gibco BRL, NY, USA); fetal bovine serum (FBS) and PBS 1X (from PAA, Canada); gentamicin, hydrogen peroxide (H_2_O_2_), MG132, propidium iodide, Nonidet P-40 (NP-40), dithiothreitol (DTT), Protease Inhibitor Cocktail and Albumin (BSA) (all from Sigma); and RNase A, (Boehringer Mannheim) and absolute ethanol from Merck.

### 2.4. Cell Fractioning

H460 (ATCC, HTB-117) cells were seeded in Flasks T-75, and were kept at 37°C and 5% CO_2_ in RPMI 1640 medium supplemented with 10% (v/v) FBS, L-glutamine plus 50 *μ*g/mL of gentamicin. The cells' fractioning was performed as described previously [15]. Briefly, the pelleted cells were resuspended in 300 *μ*L of hypotonic buffer (10 mM Hepes, pH 7.5, 10 mM KCl, 3 mM NaCl, 3 mM MgCl_2_, 1 mM EDTA, and 2 mM DTT) containing a protease inhibitor mixture from Sigma (P-8340), used at 60 *μ*L/5 × 10^6^ cells. After 15 min incubation on ice, 0,05 volumes of 10% NP-40 were added, the cells were mixed for 10 s and immediately centrifuged at 500 g for 10 min at 4°C. The supernatants were collected, labeled as cytoplasmic extracts, aliquoted, and stored at −80°C. The pelleted nuclei were resuspended in 50 *μ*L of the ice-cold nuclear buffer (20 mM Hepes, pH 7.5; 25% glycerol, 0.8 m KCl, 1 mM MgCl_2_, 1% NP-40, 0.5 mM EDTA, 2 mM DTT) containing the protease and phosphatase inhibitors as described above. Following a 20 min incubation on ice with occasional stirring, the samples were centrifuged (14,000 g, 15 min, 4°C) and the resulting supernatants were aliquoted and stored at −80°C.

### 2.5. Western Blot and Immunoprecipitation Analysis

40 *μ*g of total protein extract was applied on 7.5% to 12.5% SDS polyacrylamide gels and Western blot conducted by standard procedures [16]. The primary antibodies used were COMMD1 monoclonal (2A12), beta-actin monoclonal (AC15), heterogeneous nuclear ribonucleoproteins (hnRNP) monoclonal (4F4), anti-caspase 3, active (C8487) (all from Sigma); Bcl-2 polyclonal (C21) and Bax antibody (Santa Cruz Biotechnology), monoclonal RelA (ab95020) and polyclonal ubiquitin (ab19247) Abcam; and PARP antibody (9542) from Cell Signaling Technology. For immunoprecipitation assays, the cells were lysed in RIPA lysis buffer (1% Nonidet P40, 0.5% sodium deoxycholate, 0.1% SDS, and 0.5% BSA in PBS). The buffer was supplemented with Protease Inhibitor Cocktail and 2 mM of DTT. Immunoprecipitation assays were done using 200 *μ*g of whole-cell lysate. Rabbit polyclonal anti-RelA (ADI-KAS-TF-110) (Abcam) was used to immunoprecipitate the appropriate protein and monoclonal IgG (A-1949) (Sigma) was used as a negative control. Complexes were separated by SDS-PAGE and then analyzed by Western blot analysis. 

### 2.6. Immunofluorescence Detection of COMMD1

HT-29 (ATCC, HTB38) and MCF-7 (ATCC, HTB22) cells were seeded in 12 well plates containing cover slips (5 × 10^4^ cell/well) and were cultured in D-MEM-Glutamax, 10% fetal bovine serum, at 37°C for 24 h. Subsequently, CIGB-552 peptide was added to a final concentration of 60 and 20 *μ*M in HT-29 and MCF-7, respectively. Cells were incubated at 37°C for 5 h, then cover slips were washed with PBS, and the cells were fixed in 4% paraformaldehyde for 15 min and permeabilized in Tween 20 solution (0.2% in PBS). Blocking was performed with 3% bovine serum albumin in PBS (BSA-PBS) for 1 h. Anti-COMMD1 monoclonal antibody (2A12) (Abnova) was diluted 1 : 500 in BSA-PBS and the secondary antibody Cy3 goat anti-mouse IgG (Invitrogen) was used at 1 : 1000 dilution. All images were taken using laser confocal microscope Leica TCS SP5 and a 63x oil objective. In order to make comparable data, fluorescence images were taken using the same microscope settings (laser power, photomultiplier voltage, and offset). Four optical sections scanned at intervals of 0.3 *μ*m were taken per sample projected using Maximum Intensity Model and colored with predefined Lut (spectrum) provided in the LASAF Lite 2.6.0v software. 

### 2.7. Precipitation of COMMD1 in Pull-Down Assay

For COMMD1 precipitation, H460 (ATCC, HTB-117) cells were lysed in Triton lysis buffer (1% Triton X-100, 25 mM Hepes, 100 mM NaCl, 1 mM EDTA, and 10% glycerol) supplemented with Protease Inhibitor Cocktail and 2 mM of dithiothreitol. Pull-down assays were done using 500 *μ*g of whole-cell lysates. The biotinylated peptides L-2, L-20, and CIGB-552 (300 *μ*g) were independently incubated with 50 *μ*L of the resin streptavidin sepharose (GE Healthcare) for 1 h. Resin of streptavidin sepharose without peptides was used as a negative control. The resins were washed extensively with PBS containing 1 mM DTT. Proteins remaining bound to the resins were resuspended in 25 *μ*L of SDS sample loading buffer and separated by SDS-PAGE. COMMD1 detection was performed by Western blot using an anti-COMMD1 monoclonal antibody. The quantification of signals was carried out using ImageJ program 1.41 [17].

### 2.8. Quantitative PCR Analysis of COMMD1 Gene

H460 cells were seeded at confluence and treated with 25 *μ*M of CIGB-552 for 30 min, 2 h, and 5 h. Cells were harvested and total RNA was extracted using the AllPrep DNA/RNA/Protein mini kit (Quiagen, Valencia). cDNA synthesis from RNA using 500 ng of total RNA was done with the Quantitect Reverse Transcription kit (Quiagen, Valencia). cDNA was diluted 20-fold and 5 *μ*L was used in each quantitative PCR reaction (qPCR). Reactions were conducted in Rotor Gene 6000 equipment using ABsolute SYBR GreenQPCR kit (Abgene, Epsom). Primers sequences for reference genes: B2M, GAPDH, HMBS, ACTB, DDX5 and gene of interest COMMD1 were selected from http://primerdepot.nci.nih.gov/ database [18]. The gene expression of selected genes was conducted following MIQE recommendations [19]. Specificity of all products was verified by melting curve analysis. Reference genes were selected using GeNorm software [20]. Quantification was carried out using the ΔΔ CT method. A statistical analysis was conducted using REST software [21]. 

### 2.9. Measurement of Oxidative Stress Variables

All biochemical parameters of oxidative stress were determined by spectrophotometric methods using a Pharmacia 1000 Spectrophotometer (Pharmacia LKB, Uppsala, Sweden). Total protein levels were determined using the method described by Bradford with bovine serum albumin as standard [22]. SOD activity was determined using RANSOD kit (Randox Labs, Crumlin), where xanthine and xanthine oxidase were used to generate superoxide anion radicals (O_2_^•−^), which react with 2-(4-iodophenyl)-3-(4-nitrophenol)-5-phenyltetrazolium chloride (INT) to form a red formazan dye. SOD activity was measured by the inhibition degree of this reaction [23]. The advanced oxidation protein products (AOPP) were measured as described previously [24]. Briefly, 1 mL of samples in PBS was treated with 50 *μ*L of 1.16 M potassium iodide followed by the addition of 100 *μ*L of glacial acetic acid. The absorbance was immediately read at 340 nm. AOPP concentration was expressed as *μ*M of chloramines-T. The concentration of malondialdehyde (MDA) was determined using the LPO-586 kit obtained from Calbiochem (La Jolla, CA, USA). In the assay, the production of a stable chromophore was read at 586 nm after 40 min of incubation at 45°C [25]. Freshly prepared solutions of MDA bisdimethyl acetal (Sigma St Louis, MO, USA) were employed to generate standard curves. Ferric reducing ability of plasma (FRAP) was assayed through the reduction of Fe^3+^ to Fe^2+^ by cell lysates or ascorbic acid as reference. The Fe^2+^-2,4,6-tripiridil-s-triazine complex was detected at 593 nm [26]. All results shown are the mean of duplicates of at least three independent experiments with SE.

### 2.10. Generation of Stable H460 COMMD1 Knockdown Cell

Plasmids encoding a short hairpin control (shControl) and plasmid encoding a short hairpin targeting COMMD1 mRNA sequence (shCOMMD1) were obtained from Genecopeia (USA). Generation of H460 stable cell lines was performed with *Lentivirus* infection with the addition of 4 *μ*g/mL polybrene (Sigma). The selection was done in RPMI 1640 supplemented with 1 *μ*g/mL puromycin (Sigma) according to the manufacturer's instructions. The knockdown level of COMMD1 was determined by Western blot analysis using mouse monoclonal anti-COMMD1. Determination of the IC_50_ values was performed as described previously [1]. 

### 2.11. Cell Cycle Assay

Cells were seeded on 6 well plates during 24 h and then treated with 25 *μ*M of CIGB-552 for 24 h. The harvested cells were fixed gently by putting 100% ice-cold ethanol in freezer for 2 h. Subsequently, cells were resuspended in 300 *μ*L of PBS containing 40 *μ*g/mL of propidium iodide and 10 *μ*g/mL DNase-free RNase and incubated for 20 min at 37°C. After gating out cellular aggregates, the cell cycle distribution analysis was done on FACSCalibur flow cytometer using CellQuest software (Becton Dickinson). For each sample, at least 20,000 cells were counted and plotted on a single parameter histogram.

### 2.12. Detection of Apoptosis

Cells in early and late stages of apoptosis were detected with an Annexin V-FITC apoptosis detection kit from Sigma (041M4083). Cells were treated with CIGB-552 (25 *μ*M) and incubated for 24 h and 48 h prior to analysis. Briefly, 2.5 × 10^5^ cells were washed with PBS and adjusted in 1 × binding buffer to a concentration of 1 × 10^6^/mL. To 100 *μ*L of cell suspension, 5 *μ*L of Annexin V-FITC and 10 *μ*L propidium iodide (PI) were added and incubated for 10 min at room temperature prior to analysis. Samples were analyzed (20,000 events) using a Becton Dickinson FACSCalibur instrument. Cells that were positive for Annexin V-FITC alone (early apoptosis) and Annexin V-FITC and PI (late apoptosis) were counted.

### 2.13. Statistical Analysis

The quantitative data in this paper are represented as means ± SD. Statistical evaluation was made using the Mann-Whitney test. Differences were considered to be significant at *P* < 0.05.

## 3. Results 

### 3.1. Identification of Proteins That Interact with Peptides Derived from LALF_32–51_ Region

**Figure 1:**
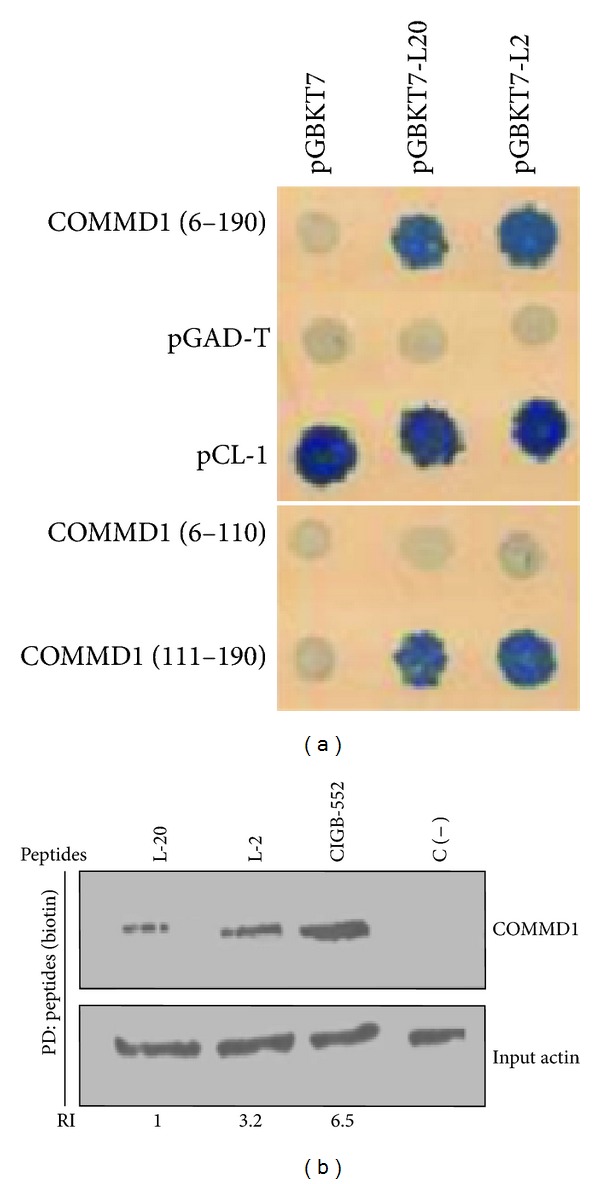
Peptides and COMMD1 interact in mammalian cells. (a) Yeast two hybrids showing interactions between full length COMMD1 and COMMD1 (111–190) prey fusions and the indicated L-2 and L-20 peptides as baits tested by interaction mating using SD Leu/Trp/His/Ade plates. pCL1 was used as a positive control of growth and the interaction between empty bait vector pGBKT7 and empty prey pGAD-T was used as a negative control. Interactions between pGBKT7 and preys constructions and pGAD-T and baits were used as a control of the interaction specificity. (b) Pull-down assay demonstrating specific COMMD1/peptides complex in H460 cells. Endogenous COMMD1 was precipitated from H460 cell lysates. The biotinylated peptides bond to streptavidin sepharose was used as bait and cell lysates as prey. The precipitated material was analysis by Western blot using antibodies directed against endogenous COMMD1. C (−) indicates streptavidin sepharose without peptide. Actin was used as input housekeeping. Relative levels of precipitated COMMD1 were determined by densitometry and normalized to actin. Ratios were depicted under each lane (RI) relative to COMMD1 levels immuprecipitated with peptide L-20. ImajeJ was used for quantification.

To identify proteins that interact with the antitumor peptides L-2 and L-20, a yeast two-hybrid screening was performed. As the peptide L-2 has shown the major antitumor activity, the plasmid pGBKT7-L-2 was selected as bait in the yeast two-hybrid screening of a human liver cDNA library to identify peptide-binding partners. A total of 10^7^ diploids were screened. Eighty-seven different positive clones were obtained. The interaction of each positive clone was verified by matting with pGBKT7 as a negative control and with constructions pGBKT7 L-2 and pGBKT7 L-20. From the initial number of clones thirty-eight positive interactions were confirmed. All positive clones were sequenced and their sequences analyzed using BLAST. Among them, a plasmid containing the sequence of COMMD1 (from amino acids 6 to 190) was identified. Interestingly, the diploid of COMMD1 with the L-20 peptide on selection plate showed a lower growth indicating a lower strength of this interaction compared with L-2 (Figure 1(a)). A second GAL-4-based yeast two-hybrid screening identified the region containing the amino acids 111–190 of COMMD1 as the potential interaction site for the peptides Figure 1(a), (bottom of the figure).

The proteolytic stability of natural peptides is one of the principal limitations for their use as drug candidates. In this study, the substitution of proline (Pro6) and leucine (Leu11) by an unnatural amino acid and blocking N-terminal ends by N-acylation from L-2 resulted in a second generation peptide named CIGB-552, which showed an increased antitumor activity *in vitro* and *in vivo *[2]. This finding suggests that the incorporation of unnatural amino acids in the sequence could improve the metabolic stability of the peptide.

To elucidate the functional significance of this chemical modification, the interaction between COMMD1 and the peptides was examined. The interaction between COMMD1 and the synthetic peptides L-2, L-20, and CIGB-552 was evaluated by pull-down analysis in human lung cancer cells H460, following Western blot with specific COMMD1 antibody. As shown in Figure 1(b), COMMD1 was detected in the peptides-pull-down precipitation confirming the existence of specific COMMD1/peptide complexes in cells that express endogenously COMMD1. Quantification of the complexes COMMD1/peptides (RI) inversely correlates with the cytotoxic activity of the peptides expressed by its inhibitory concentration (IC_50_), Table 2. The complex CIGB-552/COMMD1 increases in respect to the complex's L-2 and L-20/COMMD1 indicating that modifications done in the primary structure of the peptides increased the affinity to COMMD1 and this correlates with the increased cytotoxic activity. The results of two hybrid and pull-down experiments indicate that the interaction between the peptides and COMMD1 is specific and that the strength of this interaction may be relevant for the antitumor effect of the peptides. In addition, the interaction between CIGB-552 and COMMD1 was also confirmed in whole-cell lysates of human cancer cells of different histological origins (data not shown).

### 3.2. CIGB-552 Accumulates COMMD1 in Human Cancer Cells

**Figure 2:**
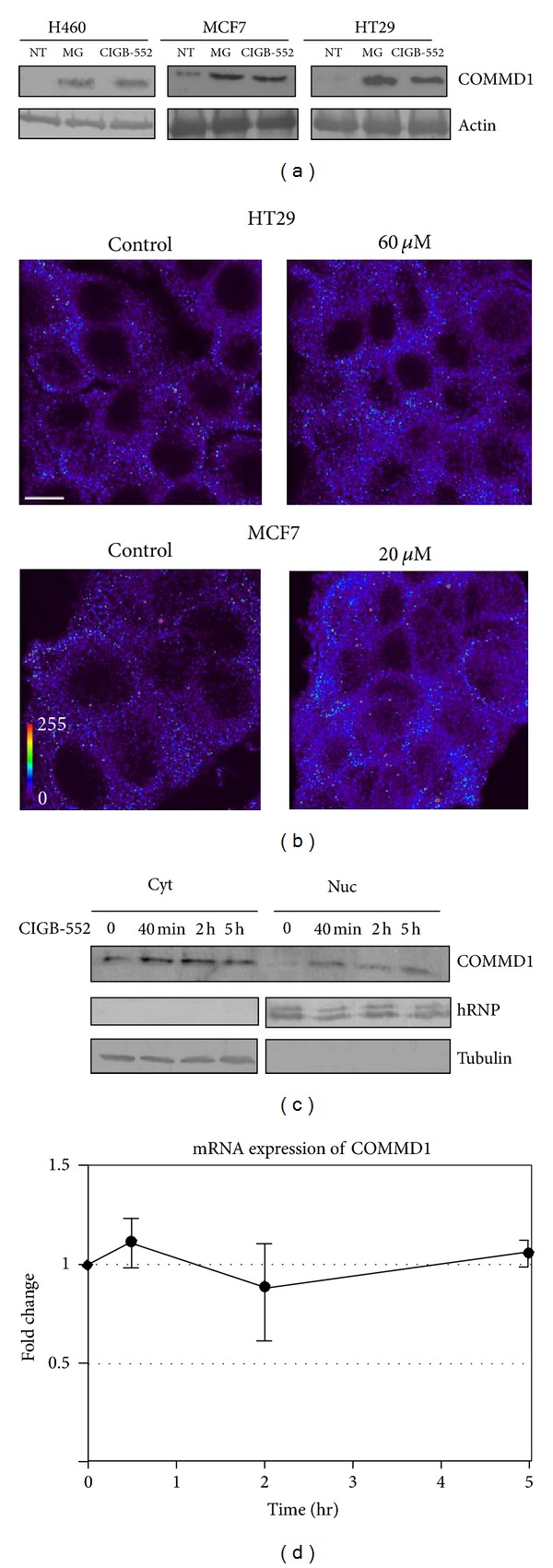
CIGB-552 promotes accumulation of endogenous COMMD1. (a) The cell lines H460, HT-29, and MCF-7 were treated with CIGB-552 (25, 60, and 20 *μ*M, resp.) or MG132 (25 *μ*mol/L) during 5 h. The levels of COMMD1 were determined by Western blot analysis of whole-cell lysates. NT untreated cells. Actin was used as a control for protein loading. (b) Cellular distribution of COMMD1 in HT-29 and MCF-7 cells following CIGB-552 treatment. Representative confocal micrographs of the duplicate samples are shown (scale bar = 5 *μ*m). Color bar (bottom left of the figure) indicate the signal strength. (c) In H460 cells, the localization of endogenous COMMD1 in the presence of CIGB-552 (25 *μ*M) at the indicated times was determined by cell fractionation followed by immunoblotting using antibodies directed against endogenous COMMD1. The protein expression of human ribonucleoprotein (hRNP) and tubulin were used as loading controls and markers for nuclear and cytosolic fractions. Nuc indicates nuclear fraction and cyt indicates cytosolic fractions. (d) COMMD1 mRNA expression determined after CIGB-552 treatment in H460 cells. mRNA expression was normalized to an expression on time point zero and shown as the mean fold induction ± SEM of three biological replicates. No differences were found at any time in the levels of COMMD1 mRNA.

Given that COMMD1 was identified as a protein that associates to the above-mentioned antitumor peptides, we studied the role of COMMD1 in the mechanism of action of CIGB-552. First, the cellular expression of COMMD1 in whole-cell lysates of human cancer cells of different histological origin was determined using Western blot analysis, and the proteasome inhibitor MG132 which induces the accumulation of COMMD1 was used as a positive control [27]. These experiments revealed an increase in the levels of COMMD1 after 5 h of treatment with the peptide. Treatment with MG132 led to the increase of COMMD1 but not at a greater extent than CIGB-552 (Figure 2(a)). The cellular accumulation of COMMD1 was also found *in situ* immunofluorescence in human cancer cells. Both cell lines assayed, MCF7 and HT29, showed the accumulation of COMMD1 after 5 h of treatment with the peptide similar to the obtained results by Western blot (Figure 2(b)). Talking together these data confirm that the peptide CIGB-552 induces the accumulation of COMMD1.

COMMD1 undergoes constitutive nucleocytoplasmic transport and its nuclear localization is needed for negative regulation of NF-*κ*B signaling [28]. Since our interest in this study is focused on the human lung cancer, the levels of the protein COMMD1 in the cytoplasm and nucleus of the H460 cells treated with CIGB-552 were evaluated by Western blot. We found a low expression of COMMD1 in untreated cells. However, in response to CIGB-552, COMMD1 was increased in the cytoplasm and nucleus of cells at 40 min following treatment, and, most interestingly, this increment remained until up to 5 h after treatment (Figure 2(c)). To examine whether the increase of COMMD1 levels is due to an increase in the RNA expression, a qPCR experiment was performed. The results showed that increased levels of COMMD1 were not accompanied by significant changes in mRNA expression of the protein (Figure 2(d)), suggesting a posttranscriptional effect of CIGB-552 on COMMD1.

### 3.3. CIGB-552 Promotes the Ubiquitination of RelA Subunit of NF-*κ*B

**Figure 3:**
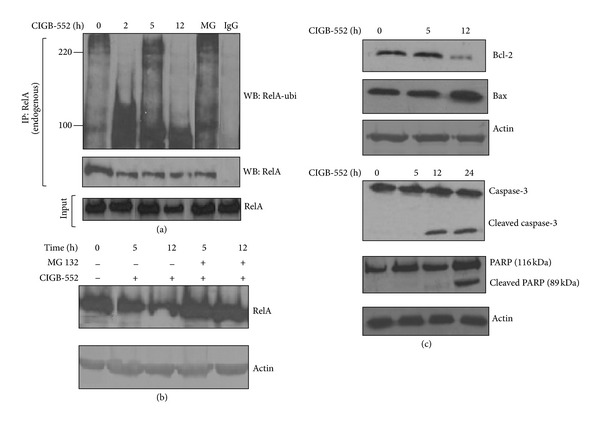
CIGB-552 induces ubiquitination and proteasomal degradation of RelA. (a) H460 cells were treated with CIGB-552 (25 *μ*M) for the times specified or MG132 (25 *μ*mol/L) for 5 h. Immunoprecipitation (IP) of ubiquitinated protein following antiubiquitin Western blot analysis (WB) show an increase of ubiquitinated forms of RelA 2 h after CIGB-552 treatment. The control of immunoprecipitation was done with anti-rabbit IgG. RelA in input samples is shown. MG indicates MG132 (positive control). (b) H460 cells were treated with either CIGB-552 (25 *μ*M) and MG132 (25 *μ*mol/L) for the indicated times. Anti-RelA immunoblot shows native protein in whole-cell extracts. The levels of RelA were increased in cells treated with CIGB-552 and MG132 with respect to the cells treated alone with CIGB-552, indicative of proteasomal degradation of RelA after CIGB-552 treatment. Actin was used as a control for protein loading. (c) H460 cells were treated with CIGB-552 (25 *μ*M) for the times indicated and Bcl-2, Bax, caspase-3, and PARP proteins were determined by Western blot analysis. Actin was used as a control for protein loading.

It has been known that overexpression of COMMD1 accelerates the ubiquitination and degradation of the RelA subunit of NF-*κ*B and decreases the activation of anti-apoptotic genes [29]. Taking into account the above data, the second question to elucidate in this study was the effect of CIGB-552 on the NF-*κ*B signaling in human lung cancer cells. The effect of CIGB-552 on the endogenous levels of ubiquitinated RelA was investigated. Immunoprecipitation of endogenous RelA using a mouse anti-RelA followed by antiubiquitin Western blot confirmed the increased amounts of ubiquitinated RelA in response to the peptide as early as 2 h after treatment, and the increment remained for 12 h after treatment (Figure 3(a)). Immunoprecipitation using a rabbit anti-IgG as negative control did not result in the recovery of ubiquitinated RelA, indicating the specificity of the recovered material. As shown in Figure 3(a), CIGB-552 as well as MG132 (used as a positive control) increased the ubiquitinated forms of RelA. To investigate whether the CIGB-552 induces ubiquitination and subsequent degradation of RelA through a proteasome-dependent process, the effect of MG132 and CIGB552 on the basal levels of the protein was tested. Treatment with CIGB-552 led to a decrease in basal levels of RelA, while protein levels were accumulated in the presence of MG132 treatment and CIGB-552. This result suggests that CIGB-552 induces ubiquitination of RelA and promotes its proteasomal degradation (Figure 3(b)).

### 3.4. CIGB-552 Regulates Apoptosis-Related Proteins in H460 Cells

Further, we assess whether CIGB-552 could modulate the expression of proteins involved in the intracellular apoptosis signaling. As shown in Figure 3(c), proapoptotic protein Bax was markedly induced, whereas Bcl-2 was significantly inhibited after 12 h of treatment with the peptide, indicating that the apoptotic effect of CIGB-552 is partly caused by upregulating the Bax/Bcl-2 protein ratio, which is a critical determinant of apoptosis. Additionally, Western immunoblotting showed a significant appearance of cleaved caspase-3 and PARP in H460 cells after 24 h of treatment with CIGB-552 (Figure 3(c)). A downstream event in the activation of the caspase-3 is the PARP cleavage. PARP helps cells to maintain their viability; the cleavage of PARP facilitates cellular disassembly and serves as a marker of cells undergoing apoptosis. Our results provide molecular evidence of apoptosis induction by CIGB-552 treatment.

The kinetics of responses to CIGB-552 was examined. We found that RelA ubiquitination occurred within 2 h of treatment (Figure 3(a)) and was associated with a nuclear accumulation of COMMD1 (Figure 2(c)). These early events had no effect on the levels of apoptotic-related proteins (Figure 3(c)). Based on our observations so far, the accumulation of COMMD1 and ubiquitination of RelA are events that precede a repression of the antiapoptotic activity of NF-*κ*B. 

### 3.5. COMMD1 Deficiency Alters Degradation of RelA Mediated by CIGB-552

**Figure 4:**
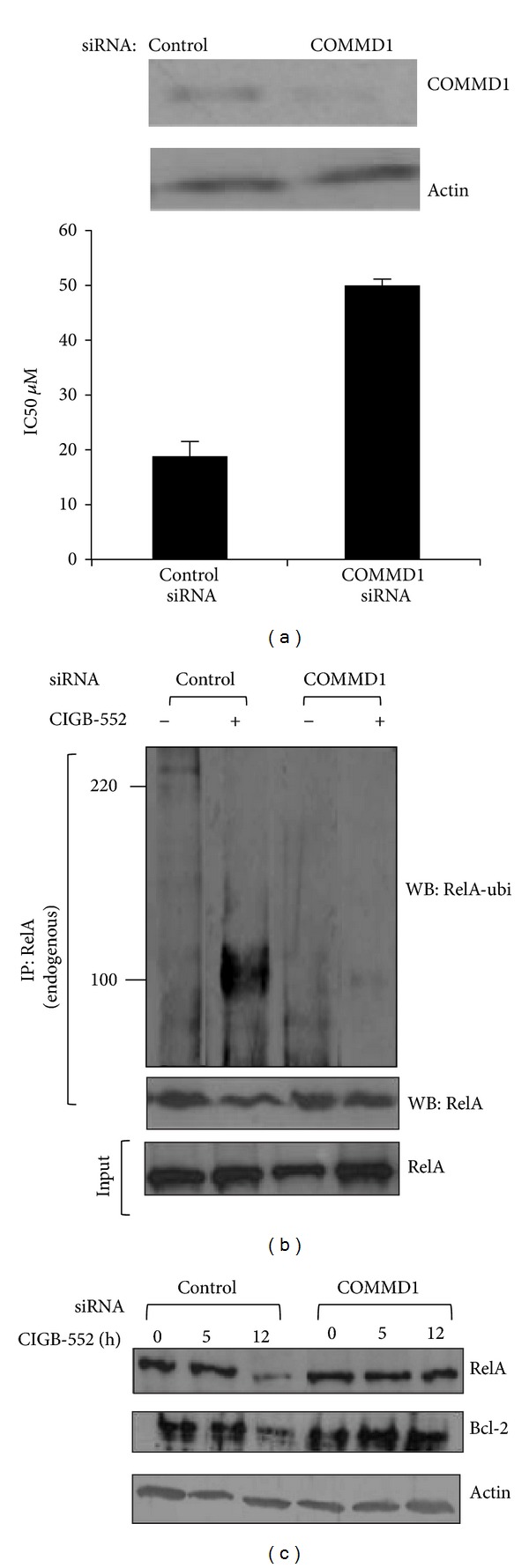
COMMD1 is involved in the antitumor activity of CIGB-552. (a) H460 cells were transfected with control or COMMD1 siRNA and then the IC_50_ values were determined using a logarithmic regression of the growth curves obtained from SRB (sulforhodamine B, sodium salt). Mean ± SD of three determinations are shown. Data were obtained from two different experiments. Top, Western blot demonstrating stable shRNA mediated repression of COMMD1 in H460 cells. Actin was used as a control for protein loading. H460 cells were transfected with control or COMMD1 siRNA and then treated with CIGB-552 (25 *μ*M) from 0 to 12 h. (b) Ubiquitination of RelA is greatly impaired in COMMD1 deficiency cells treated with CIGB-552. Anti-RelA immunoblot shows levels of ubiquitinated RelA after 5 h of treatment. (c) Repression of the levels of proteins RelA and Bcl-2 are impaired in COMMD1 deficiency treated with CIGB-552. Bcl-2 and RelA proteins levels were determined by Western blot analysis. Actin was used as a control for protein loading.

To assess if COMMD1 has a functional role in the antitumor activity of CIGB-552, knockdown of COMMD1 by siRNA was generated in H460 cell line and the cytotoxic effect of the peptide was determined. As shown in Figure 4(a), the cytotoxic activity of CIGB-552 in knockdown cells decreased in comparison to control cells. To confirm that the observed reduction in the antitumor effect of CIGB-552 in knockdown cells was not an artifact, the levels of protein were determined by Western blot. As shown in Figure 4(a), COMMD1 expression decreased in respect to control cells (top of the figure). However, the cytotoxic activity of the peptide was not completely blocked in knockdown cells. This result might be explained by the efficiency of knockdown but we do not discard that other factors could be involved in the cytotoxic activity of the peptide.

The reduced cytotoxic activity of the peptide in COMMD1 knockdown cells could be associated with decreased RelA ubiquitination and degradation. To study this possibility, the endogenous ubiquitination of the RelA levels in control and COMMD1 knockdown cells treated with the peptide was examined. As shown in Figure 4(b), COMMD1 deficiency cells resulted in lesser levels of ubiquitinated RelA when treated with the CIGB-552 in respect to the control cells. Next, we examined the levels of endogenous RelA and Bcl-2 in cytosolic extracts of control and COMMD1 deficiency cells using Western blot. As shown in Figure 4(c), COMMD1 knockdown cells showed greater levels of RelA and Bcl-2 proteins when treated with the CIGB-552 in respect to the control cells.

**Figure 5:**
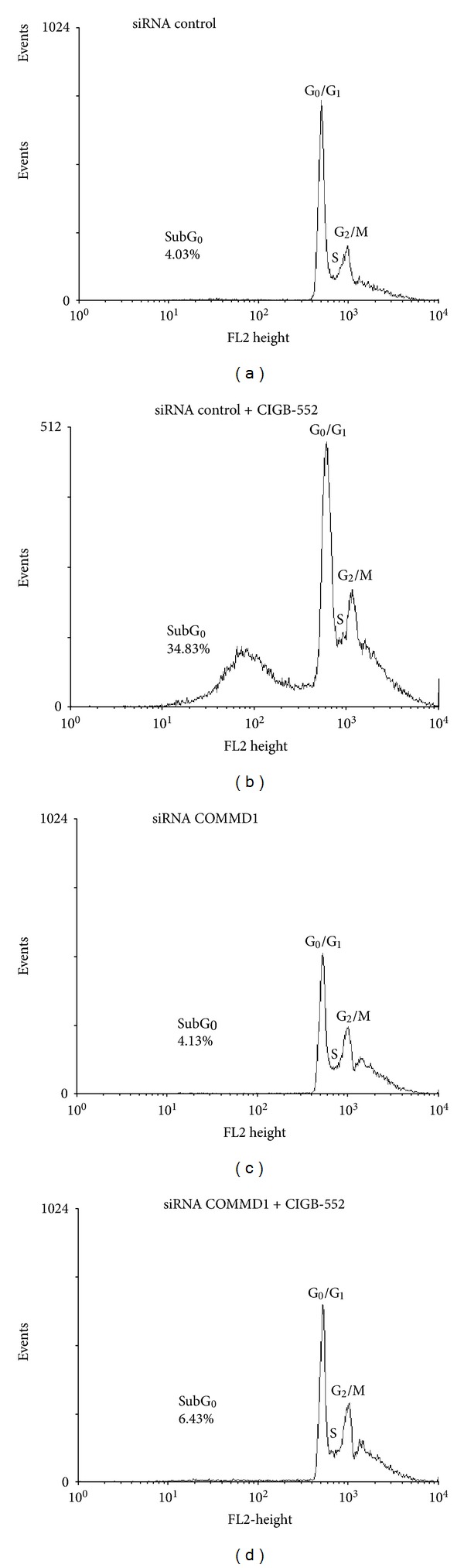
COMMD1 is involved in the apoptosis induced by CIGB-552. H460 cells were transfected with control or COMMD1 siRNA and then treated with CIGB-552 (25 *μ*M) for 24 h. The cell cycle distribution analysis was done on FACSCalibur flow cytometer using CellQuest software (Becton Dickinson). The percentage of apoptotic cells (sub-G_0_ peak) within the total cell population was calculated. Data shown here are from a representative experiment repeated two times with similar results.

**Figure 6:**
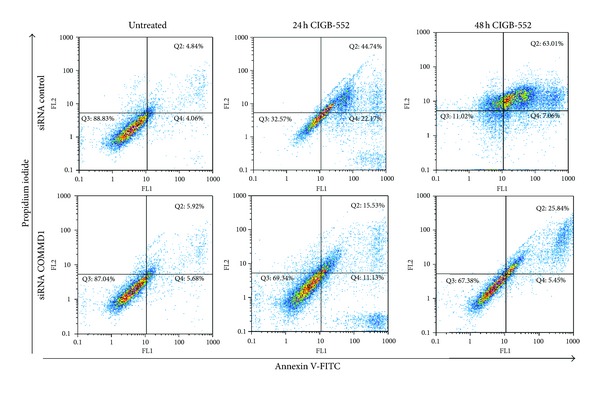
Annexin V/PI double-staining assay. Control or COMMD1 siRNA cells were treated with CIGB-552 (25 *μ*M) for 24 h and 48 h and apoptosis was examined with flow cytometry after Annexin V/propidium iodine double staining. The data shown are representative of three independent experiments with similar findings.

We have previously reported that the cytotoxic effect of the L-2 peptide involved cell cycle arrest followed by cell death [1]. Therefore, the possibility that COMMD1 might be involved in the cell death induced by CIGB-552 was explored. To elucidate this, the alteration of the cell division cycle in control and COMMD1 knockdown cells was analyzed by flow cytometry. As shown in Figure 5, control cells undergoing apoptosis after 24 h of treatment showed a significant increase in the sub-G_0_ peak. In contrast, the apoptosis induced by the CIGB-552 was blocked in COMMD1 knockdown cells. Apoptosis can be assessed by using several characteristic features of programmed cell death. One variable of apoptosis is phosphatidylserine externalization, which can be measured by Annexin V staining. The effect of the CIGB-552 on Annexin V staining was determined after 24 h and 48 h of treatment. As shown in Figure 6, control cells treated with CIGB-552 showed a significant increase in Annexin V positive cells compared with COMMD1 knockdown cells.

 Altogether, these results support the hypothesis of the effect of COMMD1 on the apoptosis induced by CIGB-552. 

### 3.6. Altered Redox Status of H460 Treated with CIGB-552

The maturation and activation of the antioxidant superoxide dismutase (SOD1) are highly regulated processes that require several posttranslational modifications. Recently, it has been described that COMMD1 is a novel interaction partner of SOD1. COMMD1 impairs SOD1 activity by reducing the expression levels of enzymatically active SOD1 homodimers late in the posttranslational maturation process of SOD1 [30]. 

**Figure 7:**
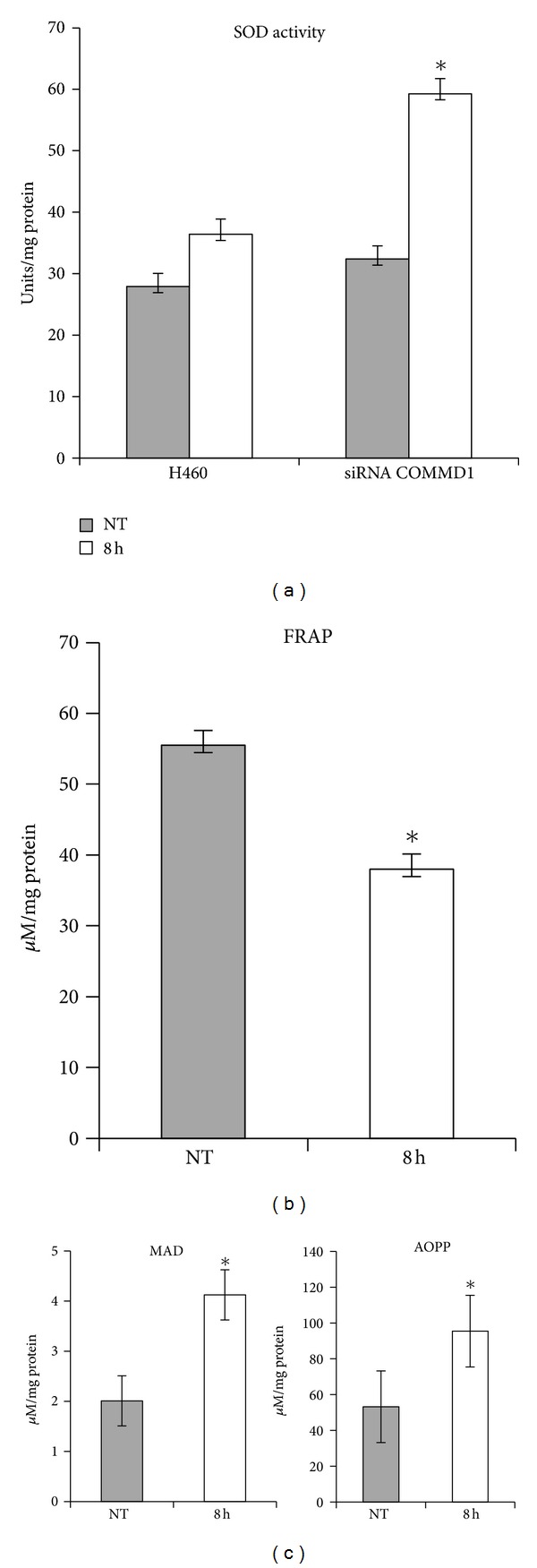
CIGB-552 modifies the redox status in lung cancer cells. H460 cells were treated with CIGB-552 (25 *μ*M) for the indicated times. (a) Activity of SOD1, H460 siRNA was used as a control of the experiment. (b) Total antioxidant capacity measured by FRAP. (c) Levels of proteins and lipids peroxidation measured by concentration of malondialdeyde (MDA) and advanced oxidative protein products (AOPP). Means ± SD (*n* = 6). *P < 0.05 statistically significant. NT indicates untreated cells.

Our previous results demonstrated that CIGB-552 induces the accumulation of COMMD1 in the cytosol. Next, we considered it important to determine the relevance of this accumulation in the enzymatic activity of SOD1, and the antioxidant capacity of the lung cancer cells. The activity of the superoxide dismutase SOD1 was measured and the total antioxidant capacity was evaluated by FRAP. Consistent with our expectation, the activity of SOD1 in knockdown cells is markedly increased compared to H460 cells after 8 h of treatment with CIGB-552 (Figure 7(a)). In line with Vonk's report [30], we also found that endogenous COMMD1 modulates SOD1 activity. As shown in Figure 7(b), the total antioxidant capacity was significantly diminished after 8 h of treatment. Next, we examined the levels of protein and lipid peroxidation, as a sign of oxidative stress damage by the formation of MDA (malondialdehyde) and AOPP (advance oxidation protein products) [31]. Concordantly, the levels of MDA and AOPP markedly increased after 8 h of treatment, likely in response to an imbalance in the antioxidant capacity Figure 7(c). Altogether, these data demonstrate that CIGB-552 might promote cell cycle arrest and apoptosis by inducing damage to proteins and lipids in lung cancer cells. 

## 4. Discussion

Our previous studies showed that the substitution of alanine in specific amino acids of the region LALF_32–51_ led to the design of peptides that lost the ability to bind LPS and exhibit a differential cytotoxic activity (L-2 and L-20). In this study, we developed the peptide CIGB-552 from the chemical optimization in the primary structure of the peptide L-2 with the purpose of reducing the elimination and biodegradation of the peptide and increasing selectivity or affinity to its potential target [32]. 

First we confirmed that the peptides L-2 and L-20 bound the region 111–190 of COMMD1 protein. Particularly a higher capacity of binding was shown by the peptide L-2 and this correlated with its higher cytotoxic activity [1]. Interestingly, our subsequent studies using the pull-down technique demonstrated that modifications in the primary structure of the peptides increased the affinity to associate COMMD1 and this correlated with increased antitumor activity. For the first time, our results identified COMMD1 as a potential target of the anticancer peptide CIGB-552 and indicated that targeting of a protein-peptide interaction may be a strategy to increase the specificity and biological activity of novel anticancer peptides derived from LALF_32–51_ region.

Recently, it has been reported that a decreased expression of COMMD1 is frequently observed in a variety of cancers and that this correlates with tumor invasion as well as with the overall patient survival. This suggests that decreased COMMD1 expression might represent a novel mechanism that confers cancer cells with invasion potential and proliferating capacity [13]. CIGB-552 was undoubtedly found to accumulate COMMD1 in cancer cells and this accumulation was not accompanied by changes in the mRNA expression. These data suggested the outstanding possibility that CIGB-552 may stabilize COMMD1 through posttranscriptional events. The basal levels of COMMD1 expression are tightly regulated by XIAP (X-linked inhibitor of apoptosis). The interaction between COMMD1 and XIAP have been mapped to the COMM domain and identified leucine repetitions within the COMM domain are required for ubiquitination and proteasomal degradation of COMMD1 [33]. Interestingly, the peptides derived from LALF_32–51_ region were found to bind COMM domain (amino acids 119–190) and, more important, our results provide the first indication that CIGB-552 could regulate the levels of COMMD1 to induce the cell death in cancer cells. The mechanism by which CIGB-552 induces the stabilization of COMMD1 is currently the issue of ongoing researches in our lab.

A central function of NF-*κ*B, in particular of RelA subunit, is the regulation of cellular growth and apoptosis [11]. Besides, the persistent activation of NF-*κ*B is a recognized contributory factor in a number of carcinomas and may provide the cancer cells with a survival advantage [34]. Recently, it has been demonstrated that ubiquitination and the degradation of RelA subunit by COMMD1-containing ubiquitin ligase is a critical mechanism of regulation of the antiapoptotic activity of NF-*κ*B and this event has considerable relevance to cancer prevention and therapy, as well as the postinduction regulation of RelA/NF-*κ*B [7, 35]. 

It was found in this study that the treatment of lung cancer cells with CIGB-552 increased the levels of the protein COMMD1 in the cytoplasm and nucleus. This observation, along with the fact that COMMD1 can repress NF-*κ*B activation, suggests a significant role for CIGB-552 in the regulation of RelA/NF-*κ*B in lung cancer cells. Our results demonstrated that CIGB-552 induces the ubiquitination of the RelA subunit of NF-*κ*B and our data also indicated that this effect promotes its proteasomal degradation. The stabilization and nuclear accumulation of COMMD1 mediated by CIGB-552 could be an attractive mechanism for the regulation of the constitutive activity of the transcription factor NF-*κ*B in cancer cells.

Consistently, our studies showed a role for COMMD1 in the cytotoxic effect of CIGB-552. H460 knockdown of COMMD1 was more resistant to the cytotoxic effect of the peptide, and this COMMD1 deficiency correlates with reduced levels of ubiquitinated RelA and apoptosis. 

Oxidative stress, involved in the etiology of cancer, results from an imbalance in the production of reactive oxygen species (ROS) and the cells' own antioxidant defenses. High levels of oxidative stress have been observed in various types of cancer cells. Accordingly, there is an aberrant regulation of redox homeostasis and stress adaptation in cancer cells and this event is associated with drug resistance [36, 37]. Here, we found that the treatment of the lung cancer cells with CIGB-552 impaired the SOD1 activity and it could be associated with the accumulation of COMMD1. Moreover, the CIGB-552 induced a failure of the antioxidant defenses and this effect was accompanied by damage to proteins and lipids. These observations suggest that CIGB-552 plays a significant role in the regulation of the redox status of cancer cells. 

## 5. Conclusions

Altogether, our results demonstrate that CIGB-552 could regulate the anti-apoptotic activity of NF-*κ*B and the oxidative stress in lung cancer cells, two biological processes involved in the survival and growth of tumor cells. 

## Figures and Tables

**Table 1:** Oligonucleotides sequences corresponding to L-2 and L-20.

Name	Sequence
L-2	CATGCACGCTAGAATCAAGCCAACCTTCAGAAGATTGAAGTGGAAGTACAAGGGTAAGTTCTGGTAA
L-2C	GATCTTACCAGAACTTACCCTTGTACTTCCACTTCAATCTTCTGAAGGTTGGCTTGATTCTAGCGTG
L-20	ATGCACTACAGAATCAAGCCAACCTTCAGAAGATTGAAGTGGAAGTACAAGGGTAAGTTCGCTTAA
L-20C	GATCTTAAGCGAACTTACCCTTGTACTTCCACTTCAATCTTCTGAAGGTTGGCTTGATTCTGTAGTG

**Table 2:** Effect of L-2 and CIGB-552 on the cell viability in different tumor cell lines.

Tumor cell line	Origin	L-2 IC_50 _(*μ*M)*	CIGB-552 IC_50_ (*μ*M)*
H460	Human nonsmall-cell lung cancer	57 ± 6	23 ± 8
H-125	Human nonsmall-cell lung cancer	75 ± 9	42 ± 6
H-82	Human small-cell lung cancer	50 ± 6	15 ± 3
LS174T	Human colon adenocarcinoma	56 ± 3	22 ± 4
MDA-231	Human breast adenocarcinoma	125 ± 3	40 ± 9
PBMC	Human mononuclear cells	234 ± 9	249 ± 6

The peptides were added to 10,000 cells in a range of concentrations from 0 to 200 *μ*M. After 48 hours of incubation, cell viability was determined by SRB (sulforhodamine B, sodium salt) assay. Finally, absorbance was measured at 492 nm, and the IC_50_ values were calculated from the growth curves.

*Mean ± SD of three determinations. Data were obtained from two different experiments.
